# Phthalate-Free Plasticization of Electrostrictive P(VDF−TrFE−CTFE) for Enhanced Actuation

**DOI:** 10.1021/acsomega.5c12507

**Published:** 2026-02-25

**Authors:** Giulio Gallucci, Andres Hunt

**Affiliations:** Department of Precision and Microsystems Engineering, Faculty of Mechanical Engineering, 2860 Delft University of Technology, Mekelweg 2, Delft 2628 CD, The Netherlands

## Abstract

PVDF-based electroactive polymer (EAP) actuators offer large field-induced strains, high compliance, and simple and scalable processing, enabling novel applications in soft robots, wearable devices, and medical devices. This work investigates how blending the poly­(vinylidene fluoride−trifluoroethylene−chlorotrifluoroethylene) [P­(VDF−TrFE−CTFE)] terpolymer with three phthalate-free plasticizers (butyryl trihexyl citrate (BTHC), 1,2-cyclohexanedicarboxylic acid diisononyl ester (DINCH), and tris­(2-ethylhexyl) trimellitate (TOTM)) affects the electromechanical transduction properties. Thin films of plasticizer/terpolymer blends were obtained via stencil printing. Film morphology (SEM), crystallinity (XRD), and mechanical and dielectric properties were investigated at different plasticizer contents, and unimorph actuators were fabricated and characterized to quantify the field-induced transverse strains. The maximum strain increased by 12.5× over the neat terpolymer in TOTM 10 wt % blends, reaching 1% at 33.2 V/μm. The largest tip deflections were achieved with TOTM 5 wt %, giving 246.6 μm at 0.1 Hz and 1.65 mm at resonance (33.7 V/μm). At a fixed field of 18 V/μm, blends with BTHC 15 wt % and TOTM 10 wt % produced 3.8 and 4× strain improvements, while DINCH 5 wt % and TOTM 5 wt % delivered 1.48 and 2.2× higher deflections. DINCH- and TOTM-based actuators withstood at least 60% higher fields than the neat terpolymer, likely due to plasticizer diffusion into the EAP film pores. These results show that the studied plasticizers can enhance transduction in P­(VDF−TrFE−CTFE), with further improvements expected by reducing film porosity, establishing optimal annealing processes and plasticizer concentrations.

## Graphical abstract

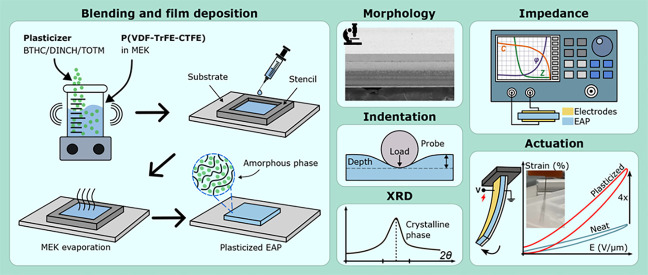

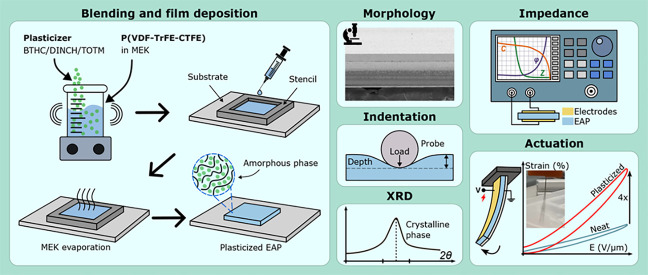

## 1 Introduction

Electroactive polymer (EAP) actuators are receiving increasing attention as versatile alternatives to conventional transducers in biomedical,^1–3^ soft-robotic,^4,5^ and haptic applications.^6,7^ PVDF-based terpolymers, such as P­(VDF−TrFE−CTFE) and P­(VDF−TrFE−CFE), are electrostrictive EAPs that exhibit high transduction response due to their relaxor ferroelectric (RFE) behavior, with reported strains and elastic energy densities of up to 9% and 1.1 J/cm^3^.^8,9^ They exhibit high dielectric permittivity, energy density, and coupling efficiency without the need of elaborate postprocessing,^8–13^ which has motivated extensive research into their implementation in actuators and devices.^14^ A common limitation to most electrostrictive polymers is the need for high electric fields to deliver large strains (>90 V/μm for 1% strain^8^). This stems from the quadratic relation between the electrostrictive strain *S* and the applied field *E*:

1 $$S=ME^{2}$$

with *M* being the apparent electrostrictive coefficient.^15^ Since *M* has been shown to depend on both dielectric permittivity ε_r_ and Young’s modulus *Y*, the electromechanical transduction in these materials can be improved via raising ε_r_ and/or lowering *Y*,^16–18^ provided that the breakdown field strength *E*_*b*_ does not deteriorate faster. These relations for the electrostrictive coefficient *M* can be written as^19^

2 $$M\propto \frac{\varepsilon_{r}\varepsilon_{0}}{Y}$$

where ε_0_ = 8.85 × 10^−12^ F m^−1^ is the permittivity of free space. Previously, altering *M* in PVDF terpolymers via ε_r_ and *Y* has been realized by blending the EAP with conductive fillers and plasticizers (see Table 1).

**Table 1:** Electromechanical Transduction Properties of Neat and Modified PVDF-Based Terpolymers

Polymer matrix	Additive	*E* (V μm^−1^)	*S^a^* (%)	*M^b^* (nm^2^ V^−2^)	*E*_*b*_ (V μm^−1^)	ref.
P(VDF−TrFE−CTFE)						
	None (neat)	200	9.0	2.25	—	^8^
	Carbon black	12	0.069	4.79	30	^20^
	Polyaniline	16	2.65	103.5	16	^21^
	DEHP	10	0.41	41	—	^15^
	DEHP	10	1.8	157*	—	^22^
	DEHP	30	1.0	11.1	207	^23^
	DINP	10	0.51	51	—	^15^
	PALAMOLL 652	10	0.21	21	—	^15^
P(VDF−TrFE−CFE)						
	None (neat)	130	4.5	2.66	—	^9^
	Carbon nanotubes	72	2.5	4.82	72	^24^
	Graphene	23	4.1	77.5	23	^25^
	DEHP	10	2.0	200.0	—	^26^
	DINP	25	1.3^c^	—	70	^27^
	DINP	20	0.35	8.75	119	^28^

a Maximum strain reported at the corresponding electric field *E*.

b *M* = *S*/*E* ^2^, unless directly reported (*).

c Strain measured under a 0.7 N preload.

Blending with conductive fillers of carbon black,^20^ carbon nanotubes,^24^ graphene,^25^ and conductive polymers^21^ (e.g., polyaniline, PANI) has shown a significant improvement in strains at low electric fields, reaching up to 4.1% at 23 V/μm.^25^ However, conductive filler incorporation drastically reduces breakdown strength, while the need for a homogeneous dispersion complicates the fabrication process.^20,25,29^

Terpolymer blends with plasticizers are much easier to prepare and have shown a smaller deterioration in the dielectric breakdown strength. Capsal et al. investigated plasticizing the P­(VDF−TrFE−CFE) matrix with DEHP (di-2-ethylhexyl phthalate) as a simpler alternative to filler-based approaches, reporting a significant increase in low-field strains (up to 2% at 10 V/μm, 15 wt % DEHP).^26^ Le et al. further showed that the decrease in breakdown strength is milder than in conductive-filler composites.^22^ Yi et al. observed similar effects in blends of P­(VDF−TrFE−CTFE) with the same plasticizer (10 wt % DEHP), reporting strains of up to 1% at 30 V/μm.^23^ The same blend in bending unimorph actuators also produced higher tip deflections (80−90% increase at 40 V/μm) compared to the neat P­(VDF−TrFE−CTFE).^30^ Della Schiava et al. blended P­(VDF−TrFE−CTFE) with 15 wt % of DINP (diisononyl phthalate), DEHP, and PALAMOLL 652 (polymeric plasticizer) and reported the highest strain increase for DINP (up to 20× increase at 10 V/μm).^15^ Terpolymers plasticized with DEHP and DINP have been studied for application in deformable mirrors,^27,31^ microfluidic pumps,^22^ morphing structures,^15^ and smart guidewires for endovascular surgery.^28,32^

As a limitation, the well-known toxicity of phthalate plasticizers^33,34^ raises concerns about their viability, especially in medical, lab-on-chip, and wearable applications. As an alternative to DEHP and DINP, modifying PVDF-based EAPs with safer alternatives that exhibit comparable plasticizing performance in polar polymers could broaden their applicability. Butyryl trihexyl citrate (BTHC), tris­(2-ethylhexyl) trimellitate (TOTM), and diisononyl cyclohexane-1,2-dicarboxylate (DINCH) are promising candidates that show no significant reproductive or endocrine effects, exhibit favorable biocompatibility and environmental profiles, and have been approved for food-contact use and established application in medical-grade polymers.^35–38^ However, among safer alternatives, only PALAMOLL 652 has been studied this far.^15^

This work investigates electromechanical transduction of P­(VDF−TrFE−CTFE) terpolymer blends with three phthalate-free plasticizers: BTHC, TOTM, and DINCH. Thin films of plasticizer/polymer blends are prepared by stencil-printing and used to fabricate unimorph bending cantilever actuators (Sections 2.1 and 2.2). The influence of plasticizer type and concentration is studied (Section 2.3) through morphological (SEM), mechanical (nanoindentation), dielectric (impedance), crystalline (XRD), and electromechanical (transduction) characterization. Section 3 presents a comparative discussion of the properties and performance of the blends, with Sections 3.6 and 3.7 focusing on their strain response and actuation behavior, respectively. Future research directions are outlined in Section 4, and the work is finally concluded in Section 5.

## 2 Material and Methods

### 2.1 Materials

The P­(VDF−TrFE−CTFE) terpolymer powder (Piezotech RT-TS) was purchased from Arkema, Germany. Three plasticizers were used in this study: butyrylcitric acid trihexyl ester (BTHC) was obtained from TCI Europe N.V. (Tokyo Chemical Industry, Belgium); 1,2-cyclohexanedicarboxylic acid diisononyl ester (DINCH) was purchased from BLD Pharmatech (Shanghai, China); and triethylhexyl trimellitate (TOTM) was acquired from Sigma-Aldrich (Merck, Germany). For the solvent, methyl ethyl ketone (MEK) was obtained from Sigma-Aldrich.

In actuator fabrication, a flexible polyethylene terephthalate (PET) film with a microporous resin coating (Novele, Novacentrix) was used as the substrate material (total thickness 140 μm). A water-based carbon black (CB) nanoparticle ink (JR700-HV, Novacentrix) was used to print the bottom electrodes (5 wt % CB content), and a 350−400 nm thick gold leaf was used for the top electrode.

### 2.2 Fabrication

#### 2.2.1 EAP Film Samples

Precursor inks of each terpolymer-plasticizer blend were prepared by first dissolving the terpolymer (10 wt %) in MEK, adding the required amount of plasticizer, and continuously stirring the solution for 6 h at room temperature (Stuart UC152 magnetic stirrer) until visually homogeneous. The resulting 12 polymer-plasticizer inks, respectively, containing 5, 10, 15, and 20 wt % of each plasticizer (BTHC, DINCH, or TOTM), were kept at 5 °C until printing.

Thin film samples of each ink were stencil-printed on different substrates as follows: (1) for nanoindentation and morphology analysis (SEM), 44.5 μm thick films were cast directly on the PET-based substrates (Novele); (2) for X-ray diffraction (XRD) measurements, 110 μm thick free-standing membranes were obtained by stencil-printing the inks on 0.2 mm thick stainless steel sheets, drying, and peeling; and (3) for dielectric characterization, 110 μm thick films were deposited on 50 μm stainless steel sheets. After deposition, the samples were dried for 12 h at room temperature to evaporate MEK. Details of the stencil-printing process can be found in previous work.^39^

The samples for characterizing dielectric properties were further coated with a 10 mm diameter gold leaf for the top electrode. A 350−400 nm thick gold leaf was placed over the EAP layer, and pressure was applied using a custom assembly consisting of a steel plate to apply pressure, a foam block to uniformly distribute it, and a thin PTFE layer to facilitate release.

#### 2.2.2 Unimorph Actuators

Unimorph cantilever actuators (Figure 1) were fabricated to study the strain response of the plasticized terpolymer films. First, carbon black (CB) bottom electrodes were printed in 10 iterations onto the PET-based substrates using an airbrush-based 3D printer.^40^ During the printing, the samples were maintained at 50 °C to promote drying and minimize splashing and were further dried at 80 °C for 6 h to ensure complete water removal. The electroactive polymer layer was then stencil-printed and dried for 6 h at room temperature, resulting in a 44.5 μm film thickness. Next, the top electrode was applied, following the same procedure as in dielectric properties characterization samples (Section 2.2.1). The outline of the 18 mm × 6 mm unimorph cantilever actuator was then cut out from the substrate using femtosecond laser micromachining (Lasea LS Lab).

**Figure 1:**
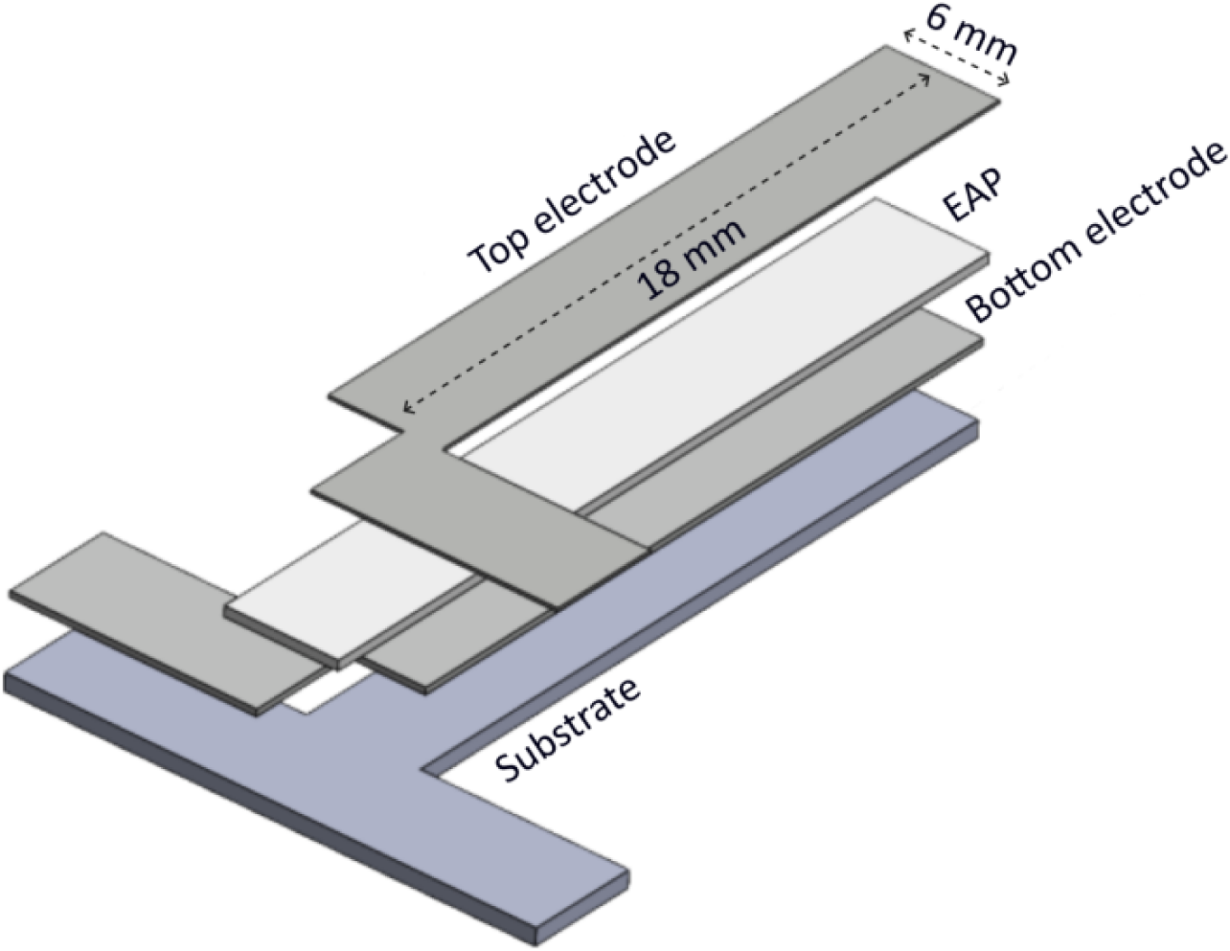
Schematic of a unimorph cantilever actuator.

### 2.3 Characterization

#### 2.3.1 Nanoindentation

The mechanical properties of the plasticized terpolymer films were evaluated using nanoindentation (Piuma, Optics11 Life), a common technique to characterize the mechanical behavior of polymeric materials.^41–43^ All measurements were conducted using a cantilever probe with a spherical tip (10.5 μm radius) and 203.4 N/m stiffness, and at least 20 indentations (maximum depth 1 μm) were performed over the surface of each 18 mm × 6 mm sample. The Young’s modulus *Y* was then extracted by fitting the Hertzian contact model to the loading segment of the indentation curves,^44^

3 $$P=\frac{4}{3}\frac{Y}{1-\nu^{2}}\sqrt{R}h^{3/2}$$

where *P* is the applied load, *R* is the indenter tip radius, *h* is the indentation depth, and ν is the Poisson’ ratio. A value of ν = 0.48 (reported for neat terpolymer)^45,46^ was used for all formulations and assumed to not vary significantly over the investigated plasticizer range (≤20 wt %). Limited variations of Poisson’s ratio at low plasticizer contents have been reported in plasticized polymers (Δν < 0.05 up to 20 wt %).^47–49^ According to eq 3, using ν = 0.45−0.50 instead of 0.48 changes the *Y* estimation by +3.6% to −2.5%.

#### 2.3.2 Dielectric Properties

Dielectric behavior of the EAPs was measured using a Keysight E5061B vector network analyzer (VNA) operated in impedance analysis mode.^50,51^ The VNA was equipped with Kelvin clip test leads (RS-PRO 123-5979) and a custom-made clamp with circular Ag contact electrodes. The disc-shaped area between the sample electrodes (Section 2.2.1) essentially forms a parallel plate capacitor that is electrically connected to the VNA by this custom clamp.

The relative permittivity ε_r_ was determined from capacitance measurements in the frequency range 30 Hz−1 MHz (room temperature) according to $\varepsilon_{r}=\frac{Ct}{\varepsilon_{0}A}$ , where *C* is the capacitance (parallel configuration), *t* is the sample thickness, *A* is the electrode area, and ε_0_ = 8.85 × 10^−12^ F m^−1^ is the permittivity of free space. All measurements were acquired at an excitation of 0.6 V amplitude in two consecutive sweeps: (i) a low-frequency sweep from 30 to 100 Hz and (ii) a higher-frequency sweep from 100 Hz to 1 MHz. The two data sets were merged by replacing the 100 Hz value with the average of the two 100 Hz measurements (difference of ≤3% for all samples).

The dielectric loss tangent (tan δ) was derived from impedance measurements as $\tan\delta =\frac{G}{2\pi fC}$ , where *G* is the conductance. The loss tangent is only presented for *f* ≥ 100 Hz since it is highly sensitive to the noise in the impedance phase at low frequencies (see Section 3.3).

#### 2.3.3 X-ray Diffraction (XRD)

The crystalline structure of the films (Section 2.2.1) was analyzed by using a Bruker D8 Advance X-ray diffractometer (Bruker Corp., Germany) configured in Bragg−Brentano geometry and equipped with a Lynxeye position-sensitive detector. A copper X-ray tube (Cu Kα, λ = 1.5406 Å) was used as the radiation source, operated at 40 kV and 40 mA. Diffraction patterns were collected in θ−2θ mode over a range of 5° to 110° (2θ), with a step size of 0.03° and a counting time of 2 s per step. X-ray diffraction data were processed by using DiffracSuite.EVA software (v7.2, Bruker AXS). Prior to analysis, background subtraction and Kα_2_ stripping were applied. The degree of crystallinity *X*_*c*_ was then evaluated from

4 $$X_{c}=\frac{A_{c}}{A_{c}+A_{a}}$$

where *A*_*c*_ and *A*_*a*_ are the integrated areas of the crystalline peak (2θ ≈ 18°) and the amorphous halo (2θ between 30° and 50°) in the XRD patterns, respectively. Crystallite size *D* was estimated using the Scherrer equation:

5 $$D=\frac{K\lambda }{\beta \cos\theta }$$

where *K* = 0.9 is the shape factor, β is the full width at half-maximum (fwhm) of the crystalline peak, and θ is the Bragg angle. The interlayer spacing (*d*) of the lattice planes associated with the crystalline reflection was calculated from Bragg’s Law:

6 $$d=\frac{\lambda }{2\sin\theta }$$

#### 2.3.4 Scanning Electron Microscopy (SEM)

Morphology of the plasticized P­(VDF−TrFE−CTFE) films and unimorph actuators was characterized by using a scanning electron microscope (JSM-6010LA, JEOL Ltd., Tokyo, Japan). Top-view and cross-sectional images were acquired at an accelerating voltage of 10 kV. Cross sections were exposed by femtosecond laser cutting (Lasea LS Lab) at reduced power and repetition numbers to minimize thermal damage to the films. Top-view SEM images of neat and plasticized terpolymer films printed on the PET-based substrate (Section 2.2.1) were analyzed using ImageJ software to quantify the cell (void) area fraction and mean cell size, expressed as the Feret diameter. Measurements were performed on three fields of view for each composition with at least 80 cells analyzed in total per sample.

#### 2.3.5 Electromechanical Transduction

Electromechanical response of the prepared films was investigated by analyzing the field-induced deflection of unimorph cantilever actuators. A block diagram and an image of the custom-built characterization setup are shown in Figures 2 and 3, respectively. The T-shaped cantilever actuators were mechanically secured by using a 3D-printed clamp (ELEGOO standard photopolymer resin 1.0, ELEGOO Mars 3), provided with embedded copper tape electrodes for electrical connections (Figure 4). Input voltages were supplied to the actuators using a high-voltage amplifier (HVA 1500/50, Smart Material Inc.), while actuator deflections were recorded in real time with a laser displacement sensor (Micro-Epsilon OptoNCDT 1900−10). The experimental workflow was managed through a custom LabVIEW 2018 interface (National Instruments), controlled from a PC via a USB-6211 (National Instruments) data acquisition unit.

During experiments, the samples were subjected to unipolar sinusoidal excitation (0.1 Hz), and the amplitude was gradually increased in 20 to 100 V increments (depending on the anticipated breakdown strength) until irreversible breakdown occurred. Samples that did not break down (i.e., breakdown strength above 1.5 kV, the capability of the high-voltage amplifier) were further characterized for dynamic performance in frequency response measurements in the 1−200 Hz frequency range at 1.5 kV amplitude. Data acquisition and storage were handled within the LabVIEW environment, and subsequent analysis was carried out using MATLAB (R2024b).

Transverse (in-plane) strain *S* in the EAP layer of the actuators was derived from the quasi-static deflections δ according to the following expression:^52^

7 $$\delta =\frac{3L^{2}}{2t}\times \frac{2AB{\left(1+B\right)}^{2}}{A^{2}B^{4}+2A\left(2B+3B^{2}+2B^{3}\right)+1}\times S$$

where *L* and *t* are the unimorph length and total thickness, *A* = *Y*_sub_/*Y*_EAP_ is the ratio of Young’s moduli, and *B* = *t*_sub_/*t*_EAP_ is the thickness ratio between the substrate and EAP layers. Design and substrate properties give *L* = 18 mm, *Y*_sub_ = 4 GPa, and *t*_sub_ = 140 μm. The elastic energy density *U*_*S*_ = 0.5*Y*_EAP_S^2^ produced by the EAP blends was further evaluated across the applied field range.

**Figure 2:**
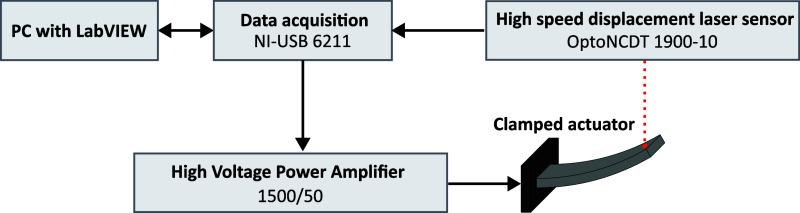
Block diagram of the actuator performance characterization setup.

**Figure 3:**
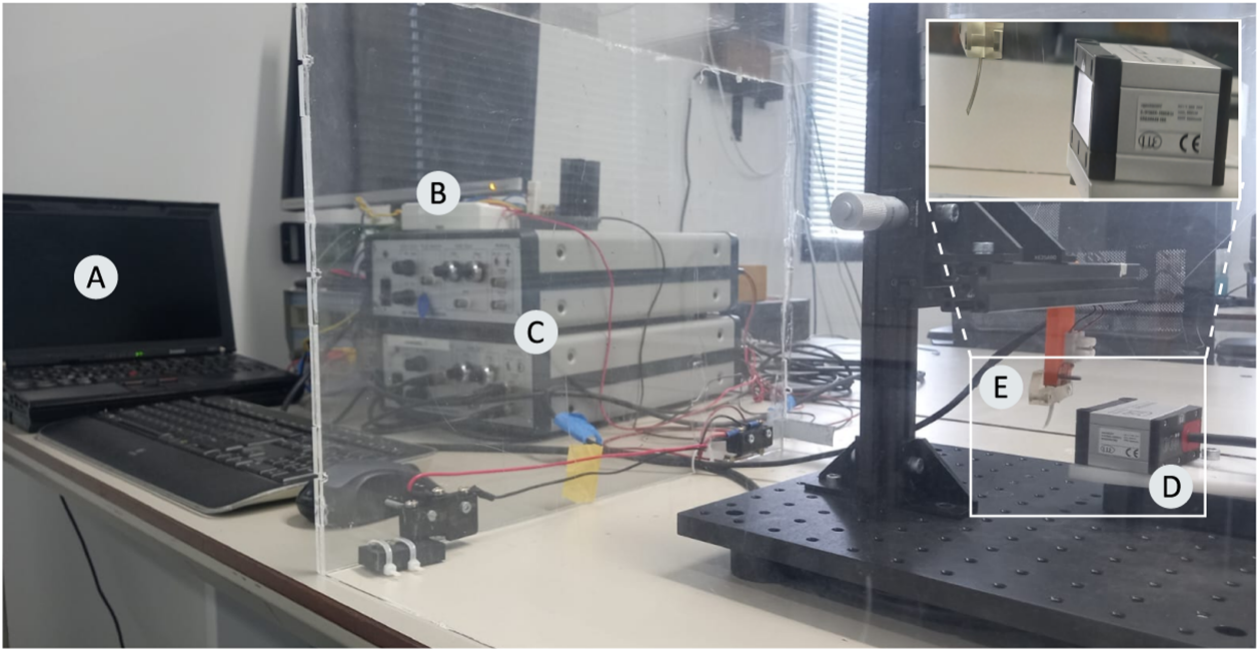
Experimental setup for quasi-static and dynamic characterization of unimorph cantilever actuators: (A) PC with the NI LabVIEW environment; (B) data acquisition system; (C) high-voltage power amplifier; (D) laser displacement sensor; and (E) clamped actuator.

**Figure 4:**
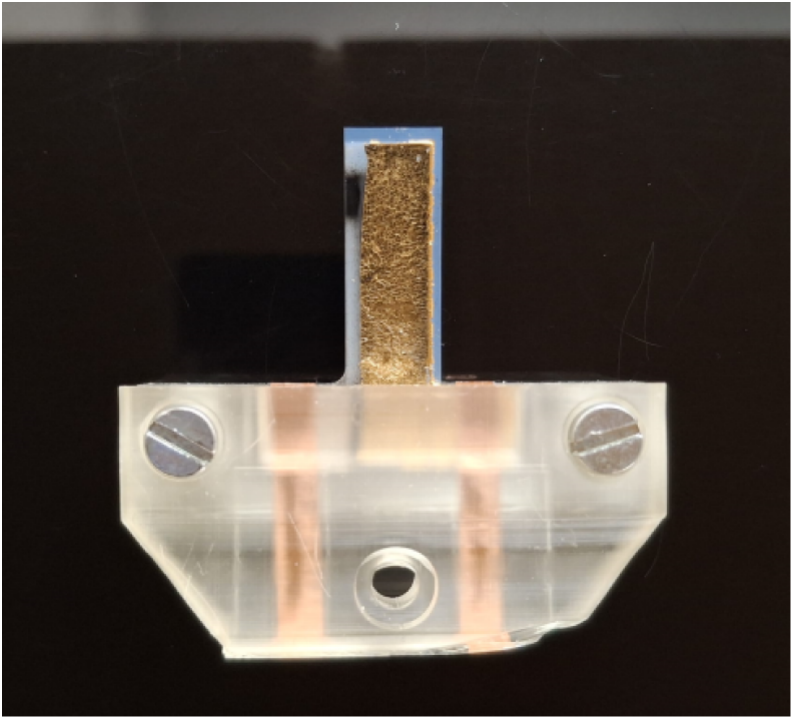
Unimorph cantilever actuator (18 mm × 6 mm) mounted into a 3D-printed clamp with embedded Cu connections for electromechanical characterization.

## 3 Results and Discussion

### 3.1 Fabrication

All inks behaved similarly during stencil printing, yielding slightly opaque films upon solvent evaporation, consistent with the porous surface observed by SEM (Section 3.5). For dielectric and XRD measurements, thicker films were fabricated (110 μm) to improve handling, ensure flatness, ease the peel-off, and minimize errors from thickness nonuniformities. Lamination of the gold leaf to form the top electrode on actuators and capacitor samples was straightforward in both neat and plasticized terpolymer samples, as the thin leaf adhered and conformed to the textured surface under light pressure, forming microwrinkles and creases that accommodate bending with low apparent stiffness (Figure 11b and Section 3.7.2).

Plasticized terpolymer films containing 20 wt % BTHC and more than 10 wt % of either DINCH or TOTM exhibited pronounced surface leakage of plasticizer after 1−2 weeks of storage at room temperature and were therefore excluded from further analysis. The observed leakage suggests limited plasticizer miscibility in the terpolymer matrix at these concentrations, leading to increased plasticizer mobility and migration to the surface during storage.^53^ Thermal postprocessing of all samples was omitted in this study to maintain a consistent baseline across all samples. Preliminary tests showed that elevated temperatures promoted plasticizer migration and initiated small cracks near fabrication defects (e.g., bubbles and dust particles) that vary with plasticizer type, content, processing temperature (80−100 °C) and exposure duration (2−10 h).

### 3.2 Mechanical Properties

Young’s moduli of the unmodified and plasticized terpolymer films were evaluated in nanoindentation experiments, as described in Section 2.3.1. The measurements are summarized in Figure 5, and typical load-indentation curves for the three plasticized terpolymer blends are given in Figure 6.

Pure terpolymer exhibited a Young’s modulus of 74.8 MPa (close to the previously reported 64.9 MPa^54^), decreasing with the addition of plasticizers (see Figure 5). For 5 wt % concentration, the Young’s moduli of all plasticized EAPs were similar (i.e., 59.3 MPa for BTHC, 57.3 MPa for TOTM, and 54.1 MPa for DINCH), while at 10 wt % concentration, the TOTM and DINCH samples showed significantly lower Young’s moduli (21.5 and 20.9 MPa, respectively) than BTHC (44.7 MPa). Softening upon plasticization results from the increased free volume and chain mobility.^55^

Load-indentation curves (Figure 6) consist of the load phase (0.55 μm/s), hold phase (1 s), and unloading phase (same rate as loading). Besides giving the Young’s moduli (from the loading segment), indentations show a load decrease during the hold segment and residual depth upon unloading (larger area under indentation curves), ascribable to the elasto-viscoplastic behavior of PVDF-based polymers that increases with plasticizer addition.^56,57^ For all samples, the load turns negative during the last portion of the unloading phase, likely due to the probe sticking to the surface of the films.

**Figure 5:**
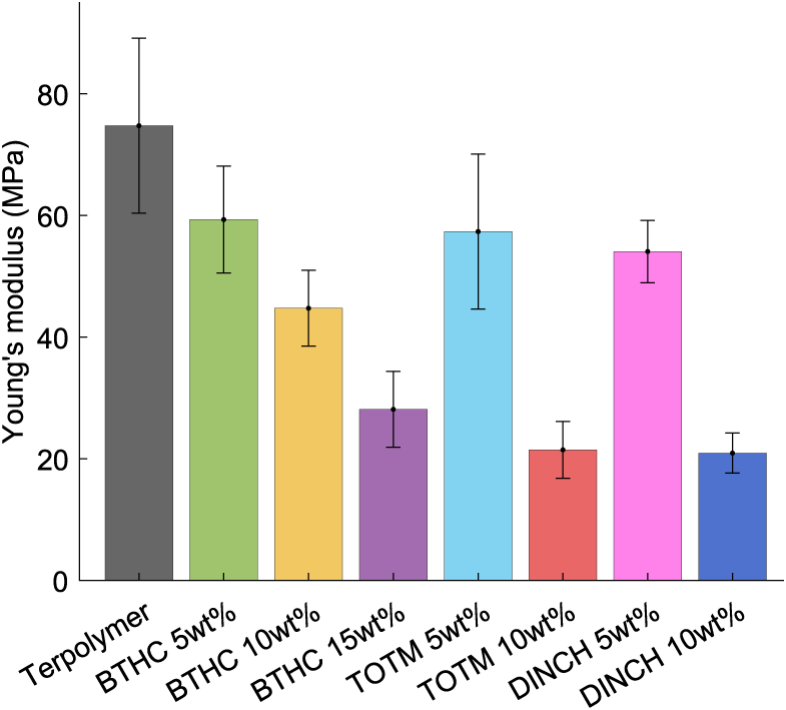
Summary of nanoindentation measurements: Young’s modulus at varying types and contents of plasticizer.

**Figure 6:**
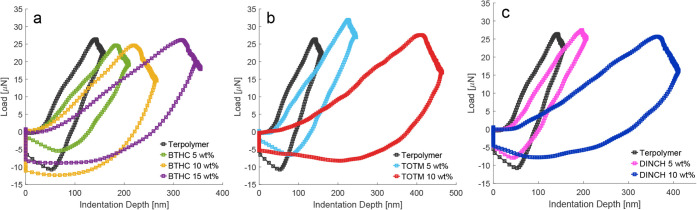
Typical load-indentation curves for BTHC (a), TOTM (b), and DINCH (c) blends.

### 3.3 Dielectric Properties

Relative permittivity (ε_r_) and loss tangent (tan δ) of neat and plasticized P­(VDF−TrFE−CTFE) are shown in Figure 7. While the relative permittivity was measured in the 30 Hz−1 MHz interval, the loss tangent measurement is overly noisy below 100 Hz due to high sensitivity to the phase angle. The different sensitivity of ε_r_ and tan δ at low frequency can be understood by writing them as functions of the impedance phase ϕ,

8 $$\varepsilon_{r}\left(\phi \right)=\frac{t}{\varepsilon_{0}A}\left[\frac{-\sin\phi }{2\pi f\left|Z\right|}\right],,\frac{d}{d\phi }\varepsilon_{r}\;=\frac{t}{\varepsilon_{0}A}\left[\frac{-\cos\phi }{2\pi f\left|Z\right|}\right]$$

9 $$\tan\delta \left(\phi \right)=-\frac{\cos\phi }{\sin\phi },,\frac{d}{d\phi }\tan\delta =\csc^{2}\phi$$

where |Z| is the impedance magnitude. Near ϕ ≈ −90°, cos ϕ ≈ 0, so noise in ϕ has a much smaller effect on ε_r_, while tan δ can be strongly affected and may become negative.

Relative permittivity decreases with increasing frequency for all samples in this study, consistent with the behavior of polar fluorinated polymers that cannot follow the rapidly changing applied field in dipole polarization.^58,59^ At 1 kHz, all plasticized blends exhibit a lower dielectric permittivity than the neat terpolymer (ε_r_ of 16.5), and ε_r_ decreases with plasticizer loading within each series (Figure 7). This trend persists above 1 kHz, and all compositions show a pronounced drop in ε_r_ near 10^5^ Hz attributable to a dipolar relaxation associated with the glass transition of the amorphous phase.^54,60^ Similar high-frequency ϵ_r_ behavior has been previously reported in phthalate−terpolymer blends.^23,61^

At low frequencies (<100 Hz), the blends show a steeper increase in ε_r_ with decreasing frequency compared to the neat terpolymer. However, only BTHC- and DINCH-plasticized samples exhibit increased permittivity at 30 Hz, with ε_r_ rising with plasticizer content up to 23 for DINCH 10 wt % and BTHC 15 wt % (vs 19.8 for the neat terpolymer). At very low frequencies (<10 Hz), an even larger increase is expected owing to enhanced interfacial (Maxwell−Wagner−Sillars) polarization upon plasticizer incorporation.^15,23,28,61^

Plasticizer modification further increases tan δ at frequencies below ∼10 kHz, and the effect becomes more pronounced with an increase in plasticizer content. At 100 Hz, BTHC produces the largest increase, with tan δ rising from 0.03 in the neat terpolymer to 0.53 in the 15 wt % blend, followed by DINCH and TOTM. The higher losses in the plasticized terpolymer samples likely reflect enhanced charge transport and interfacial polarization arising from dielectric heterogeneity between amorphous and crystalline regions.^23^

DINCH 10 wt % and BTHC 15 wt % exhibit the strongest low-frequency increase in ε_r_ and the highest losses, consistent with a larger interfacial polarization contribution than TOTM. This behavior may be influenced by differences in plasticizer structure, as TOTM is a trimellitate with a rigid benzene ring, whereas DINCH and BTHC are aliphatic and citrate ester plasticizers without an aromatic core.^62,63^ These structural differences may affect the dielectric heterogeneity in the amorphous phase.

**Figure 7:**
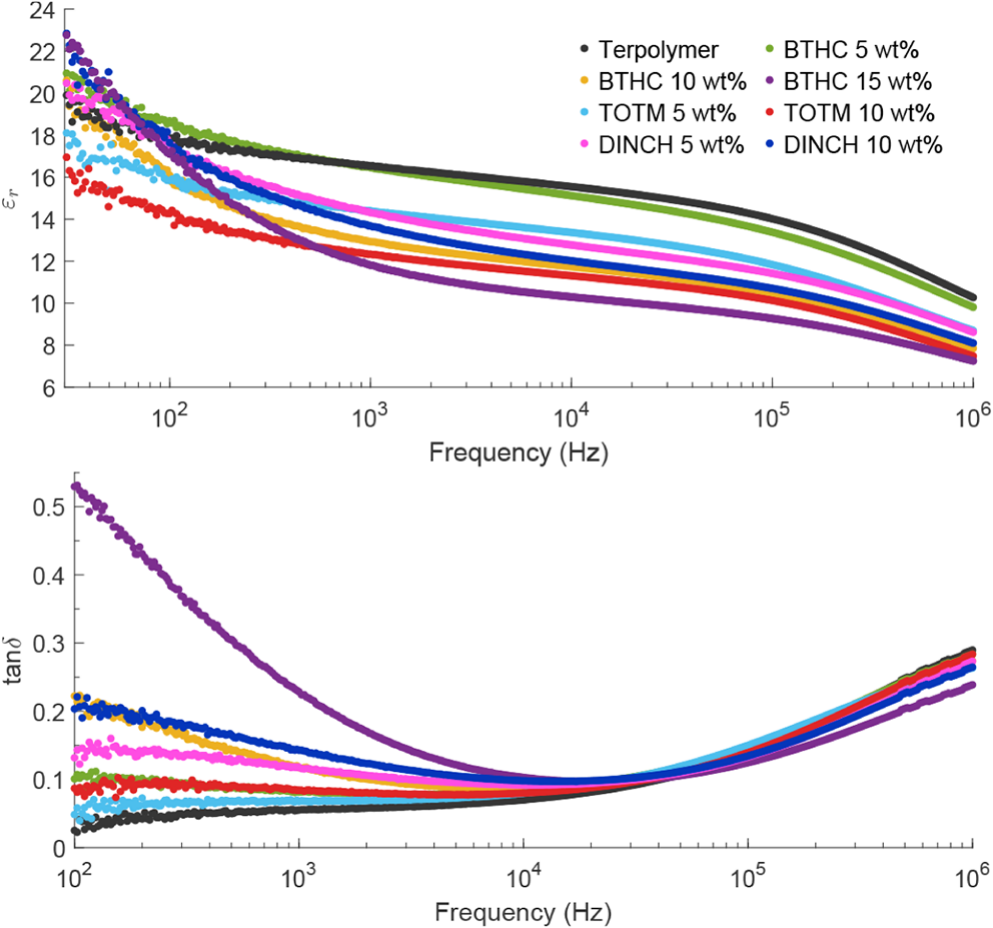
Dielectric permittivity (ε_r_) and loss (tan δ) of the terpolymer blends in the 30−10^6^ Hz and 10^2^−10^6^ Hz frequency ranges, respectively.

### 3.4 Crystalline Properties

XRD analysis was conducted according to Section 2.3.3, and Figure 8 shows the resulting patterns for the terpolymer and its blends. Table 2 summarizes the main structural parameters extracted from the patterns, including positions (2θ), crystallite size (*D*), *d*-spacing, and degree of crystallinity (*X*_*c*_). All samples exhibit a broad amorphous halo (centered around 2θ ≈ 40°) and a distinct reflection associated with the (110)/(200) planes of the orthorhombic pseudohexagonal relaxor ferroelectric (RFE) phase.^64^ The pristine terpolymer shows this reflection at 2θ ≈ 18.7°, while the plasticized samples display it between 18.5 and 18.9° with only minor variations in *d*-spacing (4.73−4.79 Å) and crystallite size (2.8−3.5 nm), indicating that the RFE crystalline arrangement is largely preserved. In contrast, *X*_*c*_ varies significantly across samples, increasing from 35% in the neat terpolymer to 39−56% in most plasticized formulations, with the exception of DINCH 5 wt % that drops to 26%.

While plasticizers typically reduce the crystalline fraction in semicrystalline polymers or leave it largely unchanged,^65–67^ the results suggest that this combination of materials and processing conditions can indirectly promote chain packing during film formation. Previous studies on plasticized PVDF-based systems have reported similar increases in crystallinity,^68,69^ which were attributed to improved chain mobility that enables more efficient polymer chain ordering during crystallization. However, for the blends studied here, quantitative confirmation of the *X*_*c*_ trends from XRD would require complementary thermal (e.g., DSC) and/or spectroscopic analysis.^70^ Different degrees of plasticizer leakage at the film surface (Section 3.1) may also add a diffuse contribution to the XRD signal and introduce variability in the estimated *X*_*c*_ values.

**Figure 8:**
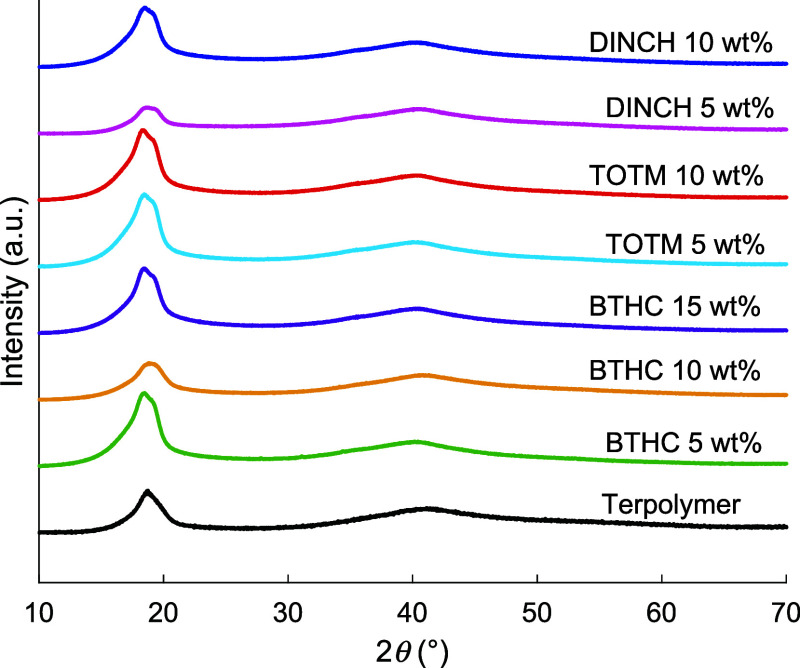
XRD patterns of the neat terpolymer and its plasticized blends.

**Table 2:** Parameters Extracted from the XRD Results (Figure 8)

Sample	2θ (°)	*X*_*c*_ (%)	*D* (nm)	*d*-spacing (Å)
Terpolymer	18.758	35	3.5	4.73
TOTM 5 wt %	18.603	55	3.0	4.76
TOTM 10 wt %	18.515	55	3.0	4.79
DINCH 5 wt %	18.810	26	3.3	4.71
DINCH 10 wt %	18.625	51	3.1	4.76
BTHC 5 wt %	18.566	56	3.0	4.78
BTHC 10 wt %	18.941	39	2.8	4.68
BTHC 15 wt %	18.605	54	3.2	4.76

### 3.5 Film Morphology

Morphology of the stencil-printed films and unimorph actuators was studied via SEM (Section 2.3.4). Figure 9 shows top-view and cross-sectional images of EAP films printed directly on the PET-based substrate (see Section 2.2.1). Top-view images were quantified in ImageJ, and the results are summarized in Table 3. Figure 10 compares top-view SEM images of the neat terpolymer film printed on the PET-based substrate and on steel, and Figure 11 shows the layered morphology of the unimorph actuators (fabricated according to Section 2.2.2).

Film surface morphologies (Figure 9) show that both the unmodified and plasticized terpolymer films exhibit a similar porous cellular surface morphology (Figure 9), with some observable interconnections to the layers below. Similar morphology has previously been reported for films of P­(VDF−HFP),^71,72^ P­(VDF−TrFE),^73,74^ and P­(VDF−TrFE−CTFE)^75^ fabricated using solvent-based methods and is commonly attributed to solvent-induced phase separation during film formation.^73,74^

As shown in Table 3, plasticization increases the cell area fraction and cell size relative to the neat terpolymer (37.5% and 1.6 μm). TOTM-modified samples change little between 5 and 10 wt % (cell size of 3.02 and 3.30 μm), while DINCH produces larger cells at 10 wt % (4.13 μm versus 2.74 μm). BTHC 5 and 10 wt % show the highest area fractions (66−67%) and the largest cells (8.3 and 6.4 μm), which decrease at 15 wt % BTHC (52% and 2.8 μm). Similar nonmonotonic pore size evolution with additive content has been previously reported for PVDF-based membranes.^76,77^ Cross-section images of the same samples (Figure 9b) further show that the polymer films densify toward the substrate.

Figure 10 compares top-view images of the neat terpolymer printed on the PET-based substrate and on steel. The film on the PET-based substrate shows markedly larger cells, which may result from differences in the film formation. Compared to steel-based films, PET-based samples are thinner (44.5 μm versus 110 μm) and are printed on a microporous coating (layer B in Figure 9b), which can affect solvent evaporation kinetics and promote phase separation. Since porosity is known to reduce the dielectric permittivity in polymer films,^78^ the smaller surface cells in films printed on steel are consistent with the relatively high ε_r_ values discussed in Section 3.3.

The spray-printed CB bottom electrodes (Figure 11a) display continuous coverage and a uniform thickness of 4 μm, corresponding to roughly 400 nm per printing iteration. The full cross-sectional view of the actuator reveals its layered structure (Figure 11b), with the thin top gold leaf electrode tightly conforming to the surface of the EAP layer, forming microwrinkles and creases that lower its apparent stiffness. Partial damage to the CB bottom electrode and localized substrate melting are attributed to the laser processing step.

**Figure 9:**
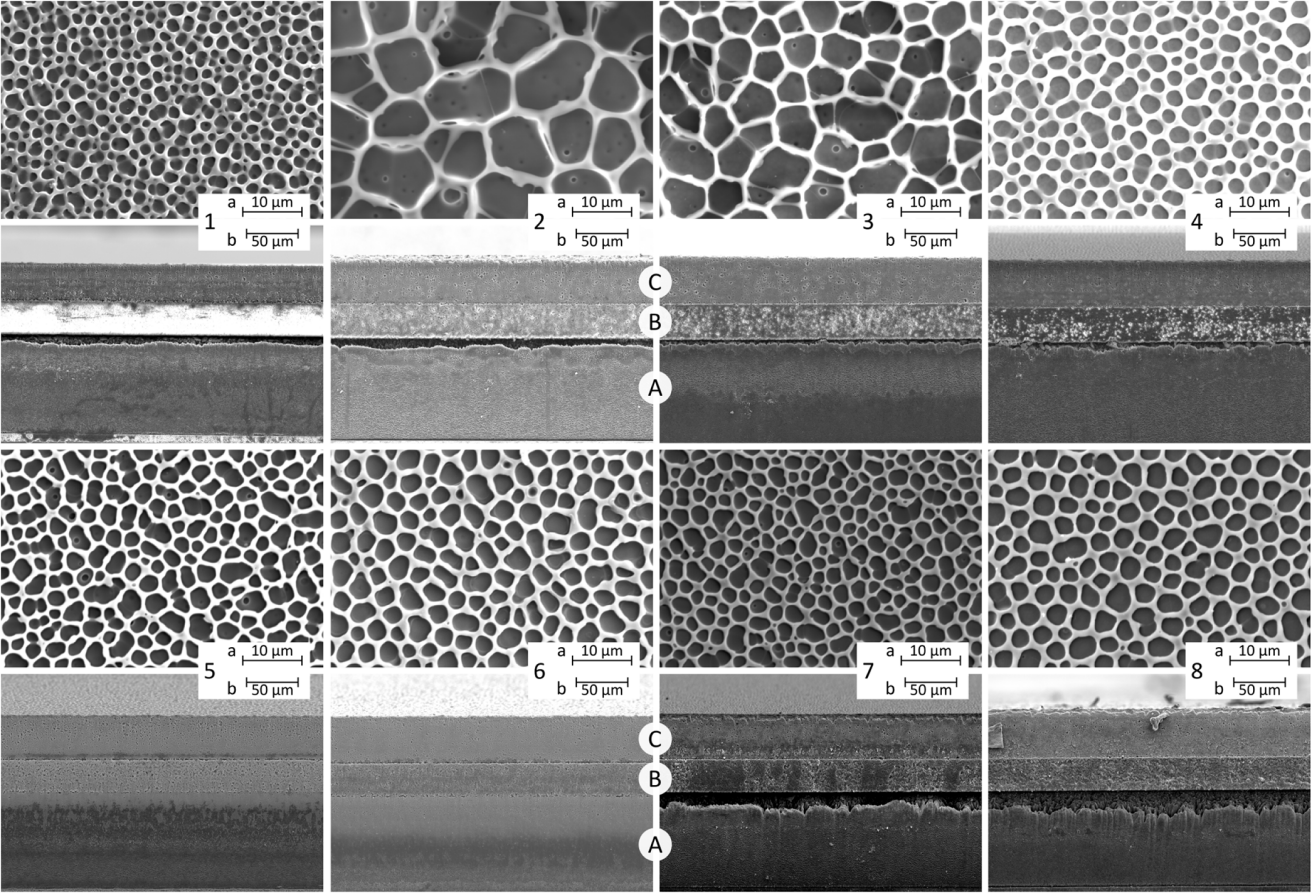
SEM top-view (a) and cross-section (b) of EAP films cast on a PET-based substrate: (1) neat terpolymer, (2) BTHC 5 wt %, (3) BTHC 10 wt %, (4) BTHC 15 wt %, (5) TOTM 5 wt %, (6) TOTM 10 wt %, (7) DINCH 5 wt %, (8) DINCH 10 wt %. (A) PET substrate, (B) the microporous resin portion of the substrate, and (C) the EAP layer.

**Table 3:** Surface Cell Area Fraction and Feret Diameter of EAP Films Printed on the PET-Based Substrate

Sample	Area fraction (%)	Feret diameter (μm)
Terpolymer	37.5 ± 5.0	1.56 ± 0.63
BTHC 5 wt %	66.1 ± 2.5	8.26 ± 3.80
BTHC 10 wt %	66.9 ± 0.8	6.35 ± 2.65
BTHC 15 wt %	52.0 ± 6.0	2.81 ± 1.06
TOTM 5 wt %	55.2 ± 3.0	3.02 ± 1.14
TOTM 10 wt %	56.9 ± 2.9	3.30 ± 1.15
DINCH 5 wt %	58.1 ± 1.2	2.74 ± 0.91
DINCH 10 wt %	60.0 ± 2.6	4.13 ± 1.15

**Figure 10:**
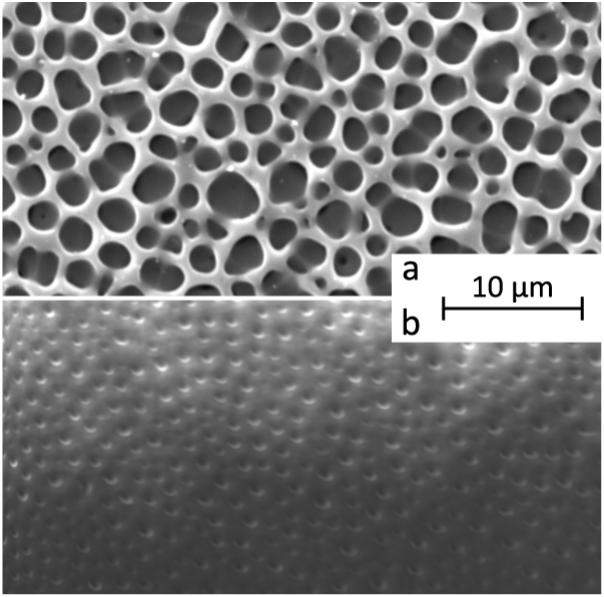
Neat terpolymer printed on a PET-based substrate (a) and stainless steel (b).

**Figure 11:**
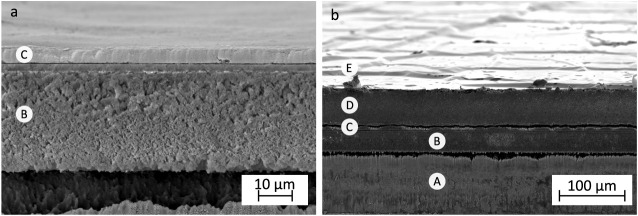
Layered structure of the unimorph cantilever actuators. CB bottom electrode on a PET-based substrate (a) and actuator cross-section (b). (A) PET substrate, (B) the microporous resin portion of the substrate, (C) the CB bottom electrode, (D) the EAP layer, and (E) the gold leaf top electrode.

### 3.6 Transduction

The tip deflections (δ) of the unimorph actuators, obtained under quasi-static excitation (0.1 Hz) according to Section 2.3.5, are presented for all pure and plasticized terpolymer actuators in Figure 12a. The field-induced transverse strains were estimated from these results according to eq 7 (see Section 2.3.5), and they are given in Figure 12b, while the associated elastic energy densities are plotted in Figure 12c. Figure 13a further shows the strain−field response for a single cycle of sinusoidal excitation at a fixed amplitude of 18 V/μm, and Figure 13b shows the field dependence of the apparent electrostrictive coefficient *M*. These results are summarized in Table 4.

**Table 4:** Summary of Electromechanical Properties of Neat and Plasticized Terpolymer Actuators

		18 V/μm			*E*_max_				
Sample	*Y* (MPa)	δ (μm)	*S* (%)	*U*_*S*_ (J/m^3^)	δ (μm)	*S* (%)	*U*_*S*_ (J/m^3^)	*M* (nm/V)^2^	*E*_*b*_ (V/μm)
Terpolymer	74.8	31.2	0.06	13.5	39.5	0.08	23.9	1.9	20.7
BTHC 5 wt %	59.3	21.4	0.05	7.5	35.1	0.09	24.0	1.6	23.8
BTHC 10 wt %	44.7	13.6	0.04	3.6	21.7	0.07	10.9	1.1	21.6
BTHC 15 wt %	28.1	46.0	0.23	75.0	46.0	0.25	87.8	6.8	18.4
TOTM 5 wt %	57.3	29.8	0.08	18.3	38.2	0.10	28.6	2.3	20.7^a^
TOTM 10 wt %	21.5	35.6	0.24	61.9	151.7	1.00	1075	8.7	>33.7
DINCH 5 wt %	54.1	46.2	0.12	40.0	210.6	0.56	848	3.8	>33.7
DINCH 10 wt %	20.9	26.0	0.17	31.5	57.1	0.39	159	5.4	29.2

a Field strength at initial actuator failure (see Figure 15c).

#### 3.6.1 Tip Displacements

Addition of BTHC in low concentrations (5 and 10 wt %) causes a decrease in the actuation response, while the 15 wt % BTHC content produced up to 1.47× higher displacements (18 V/μm). This behavior is consistent with the morphology trends in Table 3, where BTHC 5 and 10 wt % show up to 1.8× higher surface cell area fraction and 5.3× higher cell size than the neat terpolymer. Both concentrations of DINCH (5 and 10 wt %) improved the actuation, showing up to 1.48× improvement at 18 V/μm and 5.3× maximum displacements (33.2 V/μm, no breakdown). Before initial failure and recovery (see Section 3.7.2), TOTM 5% samples performed similarly to the pure terpolymer actuators, while the TOTM 10% blends exhibited up to 1.14× improvement in displacement at 18 V/μm and 3.8× maximum displacements (33.2 V/μm, no breakdown).

#### 3.6.2 Strain

Plasticizers affect transverse strains similarly to actuator tip displacements relative to the pure terpolymer (Figure 12 and Table 4). Addition of DINCH increased the maximum strain compared to that of the neat terpolymer at all investigated plasticizer concentrations, whereas modification with TOTM and BTHC showed significant improvements only at the highest concentrations. Highest maximum strains of 1.00% and 0.56% were produced by TOTM 10 wt % and DINCH 5 wt % blends (see Figure 12b) at 33.2 V/μm (no breakdown at the amplifier maximum voltage). This respectively means 12.5 and 7.5× improvement over the 0.08% (20.2 V/μm) maximum strains of the neat terpolymer. As a result, these blends also exhibited the highest elastic energy densities (see Figure 12c) of up to 1075 J/m^3^ (TOTM 10 wt %) and 848 J/m^3^ (DINCH 5 wt %), well above the maximum *U*_*S*_ of 23.9 J/m^3^ achieved by the neat terpolymer.

Figure 13a plots the strains in response to 0.1 Hz excitation at 18 V/μm field amplitude (i.e., maximum operating field of BTHC 15 wt % blend), comparing low-field strains and visualizing hysteresis. At this field strength, low BTHC contents (i.e., 5 and 10 wt %) reduce the strain response (in line with the tip deflection trends, Section 3.6.1) relative to the neat terpolymer, and TOTM 5 wt % only shows a modest strain increase (1.2×). DINCH 5 and DINCH 10 wt %, respectively, yield 2 and 2.8× higher strains, while the highest strains at 18 V/μm are exhibited by BTHC 15 wt % and TOTM 10 wt % blends, respectively, producing 3.8 and 4× higher strains than the neat polymer.

Improved material strains, therefore, do not always improve actuation strains due to the material-dependent strain−displacement ratio that depends on the Young’s moduli and thicknesses of the constituent materials (Section 2.3.5 and eq 7). This is consistent with prior work on P­(VDF−TrFE−CTFE)^54^ and P­(VDF−TrFE−CFE)^6^ unimorph cantilever actuators. As shown by Van Duong et al., unimorph tip deflection increases with increasing EAP film stress (σ = *YS*).^6^ At 18 V/μm, TOTM 10 wt % gives a lower σ (0.05 MPa) than DINCH 5 wt % (0.066 MPa), resulting in lower deflection despite the 2× higher strains (Table 4). All plasticized samples exhibit larger hysteresis loops than the neat terpolymer, ascribable to increased mechanical (Section 3.2) and dielectric (Section 3.3) losses.

#### 3.6.3 Electrostrictive Coefficient

Electrostrictive coefficients were curve-fit to the experimental strain-field relationship (described by eq 1) as shown in Figure 12b and summarized in Table 4. The apparent electrostrictive coefficient is further plotted against the field strength in Figure 13b for field strengths of 4.5 V/μm and above.

The blends with BTHC concentrations of 5 and 10 wt % showed a decrease in *M* compared to the neat terpolymer, while all other formulations improved *M*. The highest values of 6.8 and 8.7 nm^2^/V^2^ were, respectively, exhibited by BTHC 15 wt % and TOTM 10 wt %, meaning an up to 4.6× improvement. The neat terpolymer and samples with 5 wt % of BTHC and TOTM show a good fit and least variation in *M* over the field strengths (Figure 13b). Other blends show a stronger field dependence and deviation from the quadratic field-strain relation, likely originating from a field-dependent ε_r_ (see eq 2). These results are consistent with reports on DEHP/P­(VDF−TrFE−CTFE),^23^ where plasticizer incorporation has been reported to enhance field-induced strain while inducing deviations from purely electrostrictive behavior of the terpolymer matrix.

The improved *M* in the plasticized samples can be explained by an increase in permittivity (ε_r_ε_0_) and/or a decrease in Young’s modulus (*Y*), as anticipated from eq 2. As shown in Figure 14, the blends with the highest fitted *M* values (TOTM 10 wt %, DINCH 10 wt %, and BTHC 15 wt %) also exhibit the largest ε_r_ε_0_/*Y*. In contrast, the BTHC 5 and 10 wt % formulations show higher ε_r_ε_0_/*Y* yet lower *M* than the neat terpolymer, likely reflecting morphology differences (Figure 10) between films used for dielectric and XRD measurements (printed on steel) and those used for nanoindentation and actuation (printed on coated PET). Since the latter exhibit a more porous surface morphology, their effective ε_r_ε_0_ (and therefore *M*) is expected to be lower, particularly at low BTHC contents (<15 wt %), where cell size and void area fraction are highest (Table 3). The ε_r_ε_0_/*Y* ratio has previously been shown to decrease with increasing crystalline fraction derived from DSC measurements on P­(VDF−TrFE−CTFE) (*X*_*c*_ = 30.8−45.3%),^54^ whereas here no significant correlation is observed (Figure 14).

**Figure 12:**
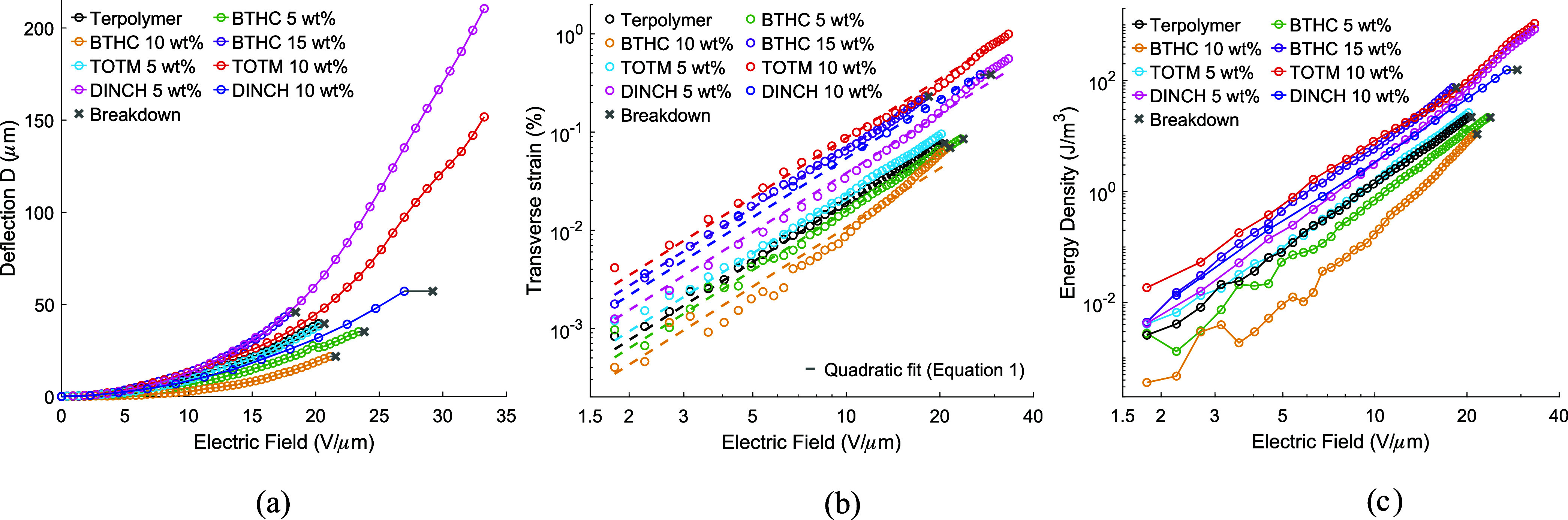
(a) Actuator deflections, (b) strains, and (c) energy densities.

**Figure 13:**
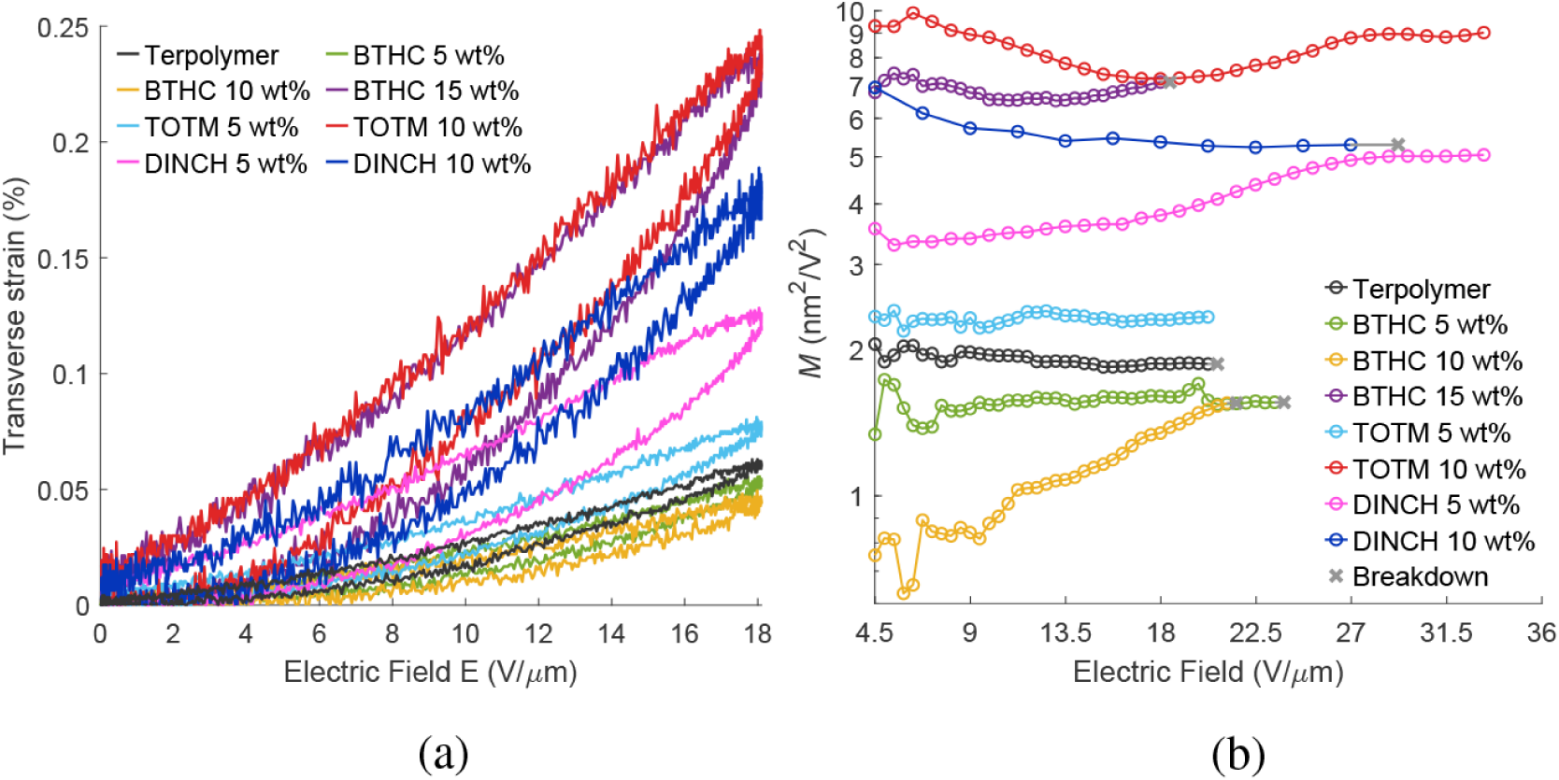
(a) Strain hysteresis and (b) the electrostrictive coefficient.

**Figure 14:**
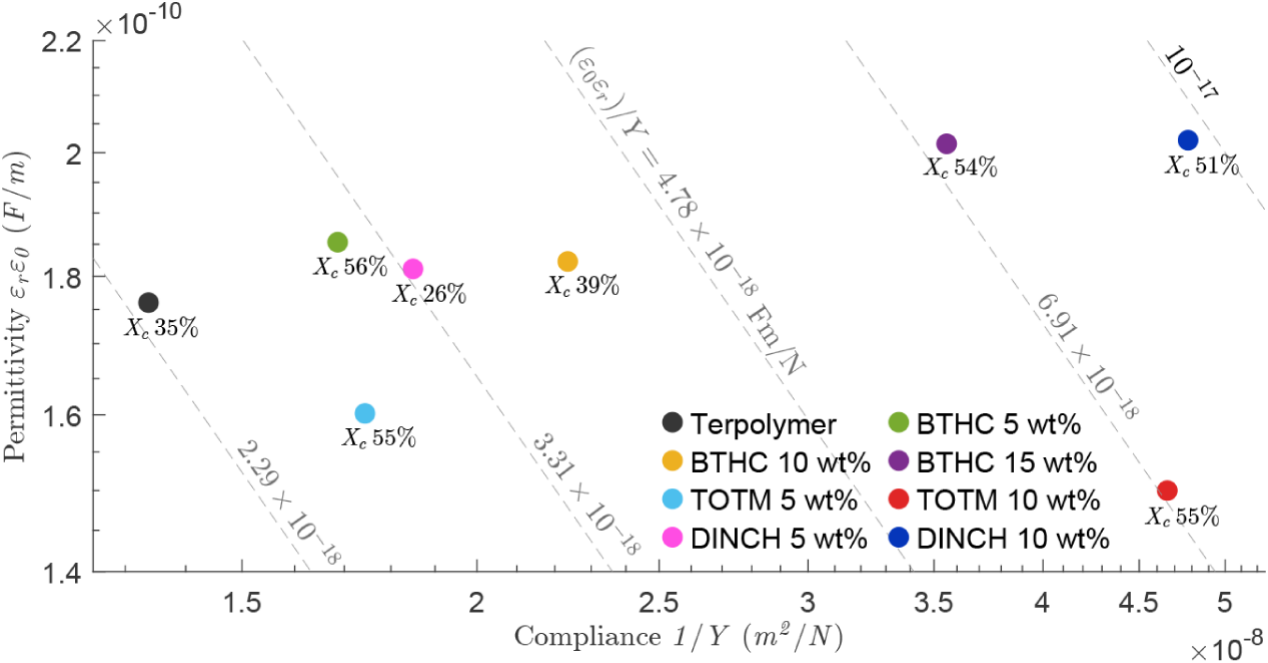
Dielectric permittivity ε_r_ε_0_ (30 Hz) versus compliance (1/*Y*) for neat and plasticized terpolymer. Dashed lines indicate a constant ε_r_ε_0_/*Y* ratio. Percentages indicate XRD-derived crystallinity *X_c_*.

### 3.7 Actuators

#### 3.7.1 Dynamics

Frequency responses of the actuators that remained operational up to the voltage amplifier limit (DINCH 5 wt % and TOTM 10 wt %) were measured according to Section 2.3.5 (1−200 Hz, 1.5 kV) and are given in Figure 15a. Highest tip displacements of up to 0.980 mm (DINCH 5 wt %) and 0.965 mm (TOTM 10 wt %) are observed at 80.6 Hz (resonance). At 1 Hz, DINCH 5 wt % and TOTM 10 wt % show tip displacements of 0.122 mm and 0.101 mm (33.7 V/μm), which are 1.73× and 1.50× lower than their respective responses at 0.1 Hz (Figure 12a). Higher deflections at 0.1 Hz are achieved at the cost of higher hysteresis in both formulations (Figure 15b), which can be attributed to enhanced viscoelastic relaxation and dielectric losses (tan δ = 0.138 for DINCH 5 wt % vs 0.086 for TOTM 10 wt % at 100 Hz, Figure 7) during the longer cycle time at quasi-static excitation.

#### 3.7.2 Self-Recovery

Two TOTM 5 wt % samples out of three showed self-recovery behavior after breaking down at intermediate field strengths. Figure 15c shows the field-deflection curves (0.1 Hz) for a TOTM 5 wt % sample that first showed visible sparks and Au electrode delamination at 20.7 V/μm (same data set in Table 4 and Figure 12). The experiment was continued in 30 min, showing 2.6× larger tip deflection (38.2 to 99.0 μm at 20.7 V/μm) and no failure at 33.7 V/μm (amplifier limit), achieving 6.2× larger maximum displacements (246.6 μm, i.e., *S* = 0.62%, *U*_*S*_ = 1100 *J*/m^3^) than the neat terpolymer. Dynamic response was measured immediately after (Figure 15a), exhibiting resonant deflections of 1.653 mm (1.5 kV, 93.8 Hz), an 8.75× improvement over quasi-static deflections (189 μm, 1 Hz). Actuation response was re-evaluated 60 days later (Figure 15c), showing slightly reduced deflections (2.2× higher than the neat terpolymer at 18 V/μm) and irreversibly failing at 25.6 V/μm.

The recovery was most probably caused by localized electrode separation at the compromised area (i.e., an electrically weak defect with lower breakdown strength), consistent with previously reported self-clearing behavior.^79^ Current-field plots (see inset in Figure 15c) indicate a current spike at the moment of failure and a slight reduction in the total resistance of the circuit after self-recovery (*ca* 2 MΩ). Lower deflections in the first run are most likely caused by current drain via a defect at the tip of the actuator, causing a voltage drop along the actuator length (high resistance of the CB bottom electrode limits the current). After the localized electrode separation (see Figure 16), this defect is removed, and EAP is exposed to a near-uniform field along the actuator length.

Since improved deflections coincided with gold leaf separation, the effect of the gold leaf on the actuator stiffness and damping was further studied. Beam vibrations were measured in response to mechanical impulse stimuli before and after applying the gold leaf on 44.5 μm-thick EAP layers (3 samples). The results showed that the addition of gold leaf increases the resonance frequency by 2.7% (2.1 Hz, SD = 0.74 Hz) and the damping ratio by 1.7% (6.8 × 10^−4^, SD = 9.1 × 10^−4^) on average. Therefore, the effect of the gold leaf on the actuator stiffness and damping is limited, as discussed in Section 3.5.

**Figure 15:**
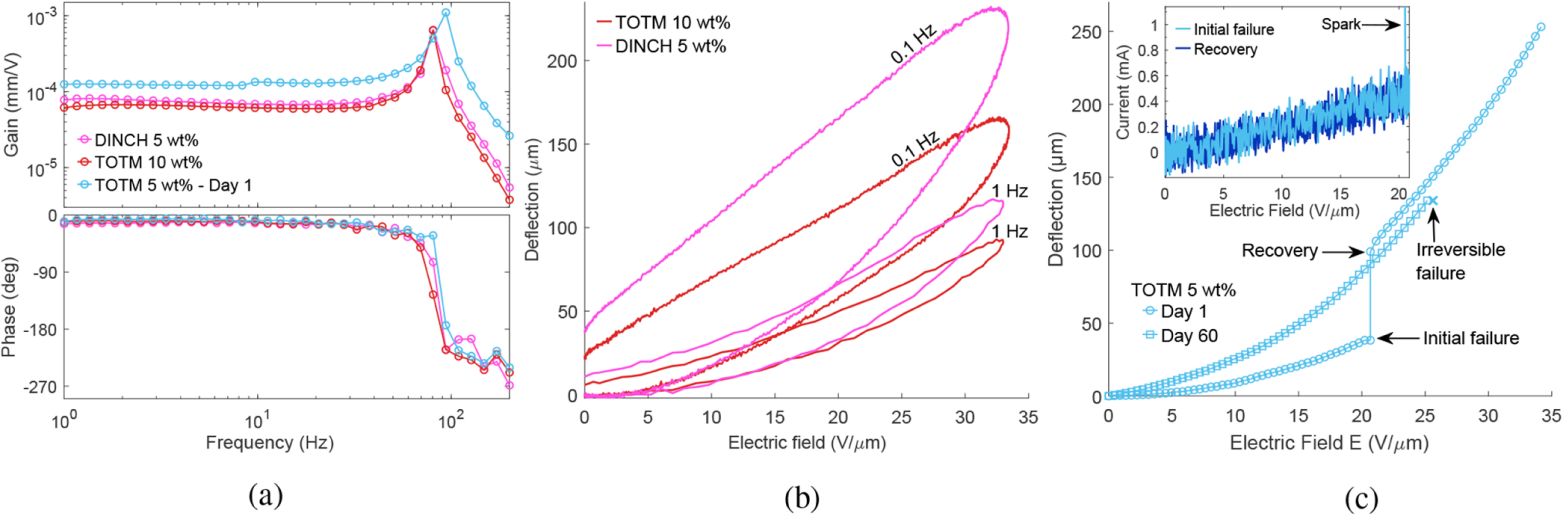
(a) Actuator dynamics, (b) deflection hysteresis, and (c) recovery.

**Figure 16:**
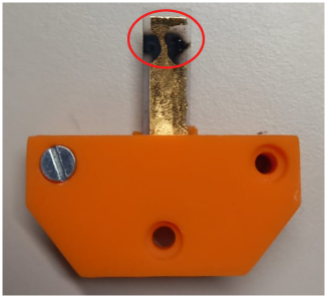
TOTM 5 wt % actuator after initial breakdown. Localized separation of the Au electrode was observed (highlighted in red).

#### 3.7.3 Breakdown in Pure Terpolymer

Breakdown of the unmodified terpolymer actuators occurred at 20.7 V/μm (see Figure 12). It manifests via localized sparking, melting, mechanical deformation, and irreversible electrical shorting of the samples. The relatively low breakdown strength is attributed to the higher film thickness (44.5 μm) and associated defects/voids (see 1a and 1b in Figure 9). As the thickness increases, the probability of critical film defects increases, and heat is less efficiently dissipated, lowering the breakdown strength.^80^ Breakdown strength *E*_*b*_ of polymer films has been shown to decrease with film thickness as *E*_*b*_ = *k* × *t* ^−*n*^, where *t* is the film thickness (normed per μ*m*), and *k* and *n* are empirical parameters.^81–83^

Breakdown strength of the pure terpolymer samples is plotted against the film thickness in Figure 17 and compared against similar samples of different thicknesses (same terpolymer and substrate, but Ag electrodes) from an earlier study.^39^ The empirical fit gives a power factor of *n* = 0.61, which is close to *n* = 0.56 previously reported for P­(VDF−TrFE−CFE).^28^ The amplitude factor *k* = 245.3 V/μm varies significantly between materials, sample geometry, and other factors.^28,83^

**Figure 17:**
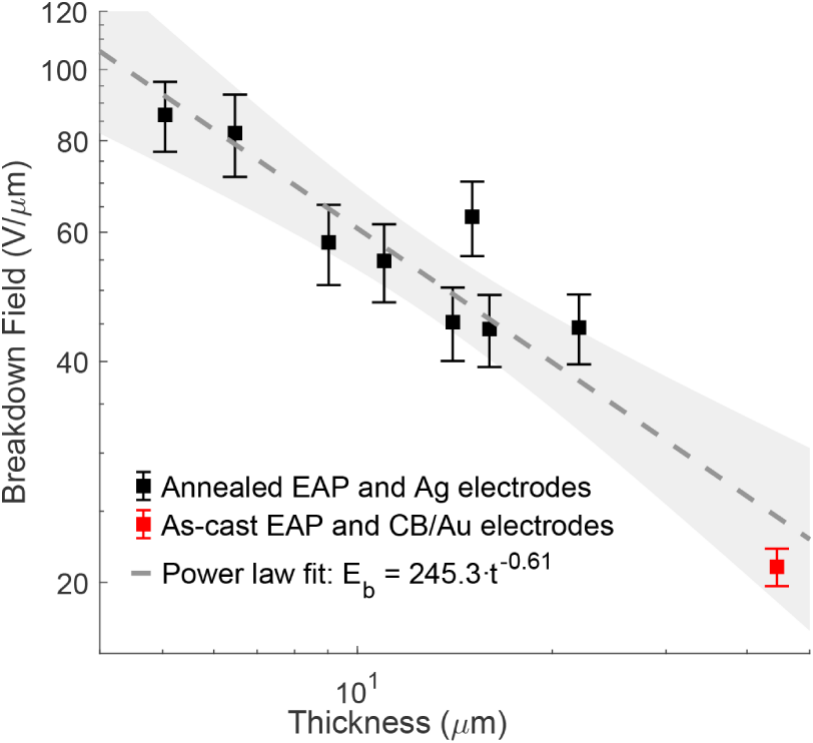
Breakdown strength vs terpolymer film thickness. Black: data reproduced from ref 39, red: pure terpolymer sample (44.5 μm), dashed line: fit, gray band: 95% confidence interval.

#### 3.7.4 Failure of Plasticized Samples

Breakdown behavior in plasticized P­(VDF−TrFE−CTFE) actuators depended strongly on both the type and concentration of the plasticizer, as can be seen in Figure 12 and Table 4. For BTHC, the breakdown field (18.4−23.8 V/μm) remained similar to that of the unplasticized terpolymer (20.7 V/μm). TOTM 5 wt % actuators first broke down at 20.7 V/μm, self-recovered to *E*_*b*_ > 33.7 V/μm, and irreversibly broke down on day 60 at 25.6 V/μm, while TOTM 10 wt % actuators did not break down at the amplifier maximum voltage (i.e., *E*_*b*_ > 33.7 V/μm). Similarly, 5 wt % DINCH samples also showed *E*_*b*_ > 33.7 V/μm, while 10 wt % samples broke down at 29.2 V/μm.

Remarkably higher breakdown strengths in the DINCH 5 wt % and TOTM 10 wt % (>60% improvement) samples are possibly caused by the higher mobility of these plasticizers than BTCH (see Section 3.1), promoting their redistribution into the EAP film pores and surface defects (Figure 9), preventing failure initiation at these sites. Similar healing mechanism has been reported by Chortos et al. in plasticized dielectric elastomer actuators.^84^

## 4 Future Research

Future work should investigate how different solvents (e.g., methyl isobutyl ketone, dimethyl sulfoxide), blend composition, and annealing processes affect film morphology, crystallinity, and the resulting electromechanical properties of the plasticized terpolymer. Studies combining dynamic mechanical analysis (DMA) with complementary techniques such as Fourier transform infrared spectroscopy (FTIR) and nuclear magnetic resonance (NMR) spectroscopy would provide insight into polymer−plasticizer interactions and solid-state miscibility across blend compositions,^85,86^ enabling formulations with optimal actuation efficiency and reliable performance.

Practical implementation in medical and microfluidic devices will further require studying how plasticizer incorporation affects actuator lifetime under cyclic loading, as well as plasticizer leachability and stability of the transduction properties across operating temperatures and environmental conditions (i.e., humidity, aging).

## 5 Conclusion

This work investigated the electromechanical transduction properties of P­(VDF−TrFE−CTFE) blends with three phthalate-free plasticizers (BTHC, TOTM, DINCH) at different concentrations. Thin films of pure and modified EAP were stencil-printed and characterized for morphology (SEM), crystalline properties, Young’s modulus, and dielectric permittivity. Unimorph bending actuators were fabricated and characterized for tip deflection, transverse strain, energy density, and electrostrictive coefficient.

Plasticization preserved the characteristic RFE crystalline reflection in XRD, while varying the film porosity, lowering the Young’s modulus, and increasing the viscoelastic losses. Dielectric measurements showed that TOTM (up to 10 wt %) reduces ε_r_ across 30−10^6^ Hz, whereas BTHC and DINCH increase low-frequency ε_r_ due to enhanced interfacial (MWS) polarization.

The maximum strains increased by up to 12.5× (1% at 33.2 V/μm) in the TOTM 10 wt % blend, while the highest tip deflections were produced by TOTM 5 wt %, showing 246.6 μm deflections at 0.1 Hz (6.2× higher than the neat terpolymer), 189 μm at 1 Hz, and 1.653 mm at 93.8 Hz (resonance, 33.7 V/μm). At a fixed field of 18 V/μm, BTHC 15 wt % and TOTM 10 wt % blends exhibited the highest strain increase (3.8 and 4× than the neat terpolymer), while the highest deflection improvements of 1.48 and 2.2× were, respectively, produced by DINCH 5 wt % and TOTM 5 wt %. TOTM- and DINCH-based actuators withstood at least 60% higher fields than the neat terpolymer, plausibly due to healing effects associated with plasticizer diffusion in the EAP film pores.

Overall, these preliminary results significantly expand the pool of plasticizers capable of enhancing the electromechanical performance of P­(VDF−TrFE−CTFE). The investigated plasticizers are less toxic than commonly reported DEHP and DINP while delivering comparable transduction improvements in the terpolymer, making these blends attractive for use in medical and wearable actuator devices. Further improvements are expected by reducing film porosity and establishing optimal annealing processes and plasticizer concentrations.
